# Editorial: Supporting the pandemic response? Implementation science in the time of COVID-19

**DOI:** 10.3389/frhs.2023.1154164

**Published:** 2023-02-23

**Authors:** Nick Sevdalis, Rachel Davis, Borsika Adrienn Rabin, Nicole A. Stadnick

**Affiliations:** ^1^ Centre for Implementation Science, King’s College London, London, United Kingdom; ^2^ Altman Clinical and Translational Research Institute Dissemination and Implementation Science Center, University of California San Diego, La Jolla, CA, United States; ^3^ Herbert Wertheim School of Public Health and Longevity Science, University of California San Diego, La Jolla, CA, United States; ^4^ Department of Psychiatry, University of California San Diego, La Jolla, CA, United States; ^5^ Child and Adolescent Services Research Center, San Diego, CA, United States

**Keywords:** pandemic (COVID19), implementation research, implementation science (MeSH), implementation theory, implementation practice

**Editorial on the Research Topic** Supporting the pandemic response? Implementation science in the time of COVID-19

In 2021, we initiated a call for papers for a Frontiers in Health Services Research Topic entitled “*Supporting the Pandemic Response? Implementation Science in the Time of COVID-19*”. The Research Topic was launched in collaboration with the 4th Annual UK Implementation Science Research Conference, organised by the National Institute for Health Research Applied Research Collaboration, South London and hosted by King's College London. The call was open to papers presented at this conference as well as submissions from individuals who did not attend the conference but were undertaking relevant research.

Building upon the conference theme, the Research Topic aimed to showcase implementation research that directly informed the response of health systems globally to the COVID-19 pandemic. The Research Topic further aimed to include research and evaluation work that is more broadly relevant. For example, research supporting long-term changes in clinical practice or public health policy that the pandemic sparked. Another example is the implementation of remote working and consultations across healthcare services.

The impact of COVID-19 on individuals, healthcare, healthcare systems and societies remain far-reaching, worldwide. Implementation science as a field has much to offer in helping to understand some of the challenges that we face now and will continue to face in the future because of the pandemic. We envisaged that the Research Topic would facilitate the emergence of an understanding within our field of what we can to do to help overcome both long-standing challenges and inequalities in care delivery – some of which were exacerbated during the pandemic. We also sought to understand and address fresh problems that also capitalize on opportunities for potential innovation at scale that the pandemic triggered. Our intention was to be self-critical of the science – asking not only how implementation science has helped the pandemic response, but also how it may have done more, what it may have done differently and what lessons can we take from this in developing the field in the future.

Here, we provide an overview of the papers included in the Research Topic. Each one of these, in a different manner, documents how implementation science has been used to address the needs and priorities created by the COVID-19 pandemic. The seven accepted manuscripts included five Original Research articles, one Brief Research Report, and one Perspective piece. These manuscripts reported on implementation efforts in response to the COVID-19 pandemic using a breadth of qualitative, quantitative, and mixed methods approaches within the United Kingdom, United States, Sweden, and South Africa.

Two papers focused on community engagement. Stadnick et al. described a pragmatic and replicable method for documenting resources for community engagement activities in health equity implementation research. Casillas et al. used mixed methods to summarize activities, initial impacts, and generalizable insights from the Share, Trust, Organize, Partner COVID-19 California Alliance (STOP COVID-19 CA), a network of 11 universities and community partners across the state of California.

Several papers offered a focus on different care pathways impacted by the pandemic and how services adapted to address the pandemic challenge. Roman et al. used a cross-sectional survey design to understand determinants of implementing remote sign language interpreting across US-based service sectors during the pandemic. Duby et al. characterized the extent to which the COVID-19 pandemic impacted implementation of an HIV, sexual, and reproductive health intervention for adolescent girls and young women in South Africa, with a specific focus on the adaptive strategies implementers employed to mitigate the pandemic effects. Pestoff et al. reported initial patient and provider implementation outcomes of telegenetic counseling in a regional Swedish healthcare system, in response to the COVID-19 pandemic limiting in-person healthcare service delivery.

Lastly, some papers focused on innovation efforts in supporting the pandemic response. Conceptually driven by an existing framework and with the motivation to support vaccination programme delivery, Pilar et al. convened a global COVID-19 implementation workgroup. The group critically applied the Implementation Outcomes Framework [Proctor et al. 2011 (1)] and reviewed implementation strategies to promote global COVID-19 vaccine implementation. Ziemann et al. examined the rapid approaches to implementing innovations of Academic Health Science Networks (AHSNs) in the English National Health System in response to the COVID-19 pandemic. AHSNs are essentially vehicles for spread and adoption of innovation, hence well-placed to respond rapidly to the fresh needs that the pandemic brought to the fore. The qualitative case study design applied in this study allowed interesting case-comparisons for managing the innovation process.

As of the writing of this editorial, across the seven accepted papers there were over 11,000 views and over 750 downloads. Visitors/readers were from North America, Europe, Asia, Australia, Africa, and South America (in decreasing order of numbers).

The Research Topic offers a collection of global case studies of pandemic-driven impacts and also innovations – in doing so, we feel that it highlights the breadth of early implementation research that was triggered by COVID-19. The included papers cover several countries and care pathways; have applied a range of different conceptual and methodological approaches to the study of implementation processes; and have offered perspectives ranging from micro (i.e., focused on individual providers) to macro (i.e., focused on entire states or countries). As more implementation studies appear in the literature motivated by the ongoing impacts of the COVID-19 pandemic, we would call upon our colleagues to report on the sustainability and spread of pandemic-driven innovations, both where they were originally implemented and beyond; and to consider and analyse the factors and strategies that are associated with success or failure of such innovations in the medium- to longer-term. Reporting on naturally occurring experiments during the pandemic and to-date may offer a fruitful and cost-effective approach to study design that generates learning and hypotheses for further controlled investigations – thereby driving the field forward.
